# Surgical Treatment Outcomes of Gynecologic Cancer in Older Patients: A Retrospective Study

**DOI:** 10.3390/jcm12072518

**Published:** 2023-03-27

**Authors:** Kyeong A So, Seung-Hyuk Shim, Sun Joo Lee, Tae Jin Kim

**Affiliations:** Department of Obstetrics and Gynecology, KonKuk University Hospital, Konkuk University School of Medicine, Seoul 05030, Republic of Korea

**Keywords:** gynecologic cancer, geriatric oncology, cancer, surgical treatment

## Abstract

This study aimed to evaluate oncologic characteristics and surgical outcomes in older patients with gynecologic cancers. This retrospective study included patients aged ≥65 years who were diagnosed with gynecologic cancers and underwent surgical treatment between 2005 and 2020. We reviewed the medical records for age at diagnosis, body mass index, American Society of Anesthesiologists score, comorbidities, postoperative complications, cancer stage, histologic type, surgical treatment, postoperative outcome, and survival rate. Data were compared between groups according to the age at the time of diagnosis: <75 years (young-old) and ≥75 years (old-old). In total, 131 patients were identified: 53 (40.5%) with ovarian or primary peritoneal cancer (OC), 44 (33.6%) with endometrial cancer (EC), 30 (22.9%) with cervical cancer, and 4 (3.1%) with leiomyosarcoma. The patients’ mean age was 70 (range, 65–83) years; 106 (80.9%) were young-old and 25 (19.1%) were old-old. Postoperative complications occurred in 19 (14.5%) patients. Four patients died within six months after surgery, and three died because of disease progression. There was no difference in the survival rates between the two groups among those with OC and EC. Older patients with gynecologic cancers showed good surgical outcomes and tolerable postoperative complications. Therefore, we can safely offer surgical treatment to older patients.

## 1. Introduction

The proportion of older patients aged ≥65 years is rapidly increasing, with an estimated proportion of 20% of the world’s population by 2050, and the proportion includes approximately 60% of patients with cancer [1,2]. Gynecologic cancers are common among older women. Almost 50% of ovarian cancer (OC) cases are in women aged >65 years, and endometrial cancer (EC) is diagnosed in older women with a mean age at diagnosis of 68 years [3,4]. Cervical cancer (CC) accounts for a quarter of the cases occurring after the age of 65 years [5].

Surgical management is the primary treatment for patients with gynecologic cancer. The primary treatment for OC and EC is a staged surgery, which includes total hysterectomy, bilateral salpingo-oophorectomy, and lymphadenectomy. In addition, surgical treatment is primarily considered for early-stage CC up to stage IIA. However, in the case of older patients with comorbidities such as hypertension, stroke, and heart disease, doctors and patients may hesitate to consider surgery because of concerns about complications and the safety of the surgery [6,7]. Previous studies have reported that the rate of surgical treatment in older patients is significantly lower than that in their younger counterparts [8,9,10]. Older patients with OC commonly receive adapted treatment and are less frequently treated with the standard guideline than younger patients [11]. In advanced-stage EC, older patients (>75 years) underwent surgery less frequently than young patients (<55 years) (*p* < 0.001) [9]. Similar results were noted in early-stage CC; the surgical treatment rates were 82% in patients aged <50 years and 55% in those aged 70–79 years [10]. Despite the poor prognostic factors of older patients, previous studies have reported that they are less often treated with lymphadenectomy, radiotherapy, and chemotherapy than their younger counterparts [12,13].

It is questionable whether the intensity of surgical treatment adversely affects the prognosis of older patients with gynecologic cancers and whether the treatment strategies need to be changed according to age. Therefore, this study aimed to investigate the surgical outcomes in older patients with gynecologic cancers and compare the surgical outcomes and survival rates according to age groups.

## 2. Materials and Methods

### 2.1. Study Design and Population

This study retrospectively investigated 131 patients aged ≥65 years who underwent primary surgical treatment for gynecologic malignancy between 2005 and 2020 at Konkuk University Hospital, South Korea. Patients who did not undergo surgical treatment or had incomplete medical records were excluded from the study.

After obtaining approval from the Institutional Review Board (number 2022-05-016-001), we collected the following data: age at diagnosis, body mass index, American Society of Anesthesiologists Physical Status Classification System (ASA class), previous medical illness and surgical history, laboratory results, operative time, estimated blood loss, blood transfusion, pathological results, time from surgery to first diet, follow-up duration, and survival outcome. The ASA class was evaluated for medical comorbidities before the surgery and was usually graded by an anesthesiologist. The ASA classes range from 1 to 6, with a lower score indicating a healthier status [14]: ASA class 1, healthy patients; ASA class 2, mild systemic disease including obesity and well-controlled hypertension (HTN) or diabetes mellitus (DM); ASA class 3, severe systemic disease including active hepatitis, implanted pacemaker, and poorly controlled HTN or DM; class 4, severe systemic disease that is a constant threat to life; class 5, a moribund patient who is not expected to survive without operation; and class 6, a declared brain-dead patient [14]. Herein, HTN was separated from other cardiovascular diseases because it is common in geriatric patients. Angina, cardiac arrhythmia, and heart failure were covered by cardiovascular disease.

### 2.2. Primary Outcome

The surgical outcomes included intra- and postoperative outcomes. The estimated blood loss was usually calculated by anesthesiologists who were attending the surgery. The postoperative outcomes were defined as any unexpected symptoms until 30 days postoperatively, and interventions to treat these complications were investigated. For the comparison of postoperative complications and survival outcomes, patients were classified into two groups according to age at the time of diagnosis: young-old, <75 years, and old-old, ≥75 years.

### 2.3. Statistical Analysis

The continuous variables were analyzed using the independent *t*-test and analysis of variance. The categorical variables were analyzed using the chi-square or Fisher’s exact test. Kaplan–Meier analysis and log-rank tests were used to compare the overall survival between the age groups. Data were analyzed using SPSS version 26 (IBM Corp., Armonk, NY, USA). A *p* value < 0.05 was considered statistically significant.

## 3. Results

During the study period, 251 patients aged 65 years or older were diagnosed with gynecologic cancer. Among them, 120 patients who did not undergo surgical treatment and two patients with incomplete medical records were excluded from the study analysis. The clinical characteristics of the study population are summarized in Table 1. The patients’ mean age was 70 years (range, 65–83 years). Forty-five (34.4%) patients had a history of abdominal surgery, and ninety-three (70.1%) patients had at least one medically diagnosed illness. Among them, 87 patients had one or more major comorbidities including hypertension, diabetes mellitus, previous malignancy, cardiovascular disease, cerebrovascular disease, asthma, thyroid disease, and renal disease. The most common illness was HTN (51.9%), followed by DM (21.4%). Ten of the patients had a history of cancer. According to gynecologic cancer types, 53 patients (40.5%) had OC, 44 (33.6%) had EC, 30 (22.9%) had CC, and 4 (3.1%) had leiomyosarcoma.

Analysis of clinical characteristics according to the gynecologic cancer type was performed (Table 2). In the OC group, the mean age of the patients was 71.1 years. Thirty-five (66%) patients were diagnosed at an advanced stage (stages III and IV). Histologically, the serous and non-serous types were observed in 34 (64.2%) and 19 (35.8%) patients, respectively. Optimal debulking surgery, which means no gross residual disease, was achieved in 36 patients (67.9%). Adjuvant chemotherapy was administered to 39 (73.6%) patients. In the EC group, the patients’ mean age was 69.9 years. Endometrioid and non-endometrioid histologies were observed in 27 (61.4%) and 17 (38.6%) patients, respectively. Lymphadenectomy was performed in 33 patients (75.0%), and adjuvant treatment was administered to 32 (72.7%) patients (11 patients in chemotherapy, 15 patients in chemotherapy, and 6 patients in chemoradiation therapy). In the CC group, the mean age of the patients was 68.8 years. Squamous cell carcinoma (SCC) and non-SCC histologies were observed in 26 (86.7%) and 4 (13.3%) patients, respectively. All of the non-SCC histologies were adenocarcinoma. A radical hysterectomy was performed in 66.7% of patients, while others underwent simple hysterectomy. Adjuvant treatment was administered to 14 (46.7%) patients (13 patients in concurrent chemoradiation therapy and 1 patient in radiation therapy). When compared by cancer type, the age at diagnosis did not show a significant difference (*p* = 0.081). The cancer stage was significantly more advanced in the OC group than in the EC and CC groups (*p* < 0.001 and *p* < 0.001, respectively). More patients in the advanced stage had EC than CC (*p* = 0.028). A laparotomy was performed significantly more often in the OC group than in the EC and CC groups (*p* < 0.001 and *p* = 0.001, respectively). A lymphadenectomy was performed in more than half of the patients among all cancer types. Four patients with leiomyosarcoma were diagnosed at a mean age of 70.1 years. All patients underwent a debulking surgery by laparotomy. The histopathologic results were shown one patient in stage IB, two patients in stage IIIA, and one patient in stage IVA. There were no postoperative complications and the average hospital stay was 10.1 days. The stage I patient who received adjuvant chemoradiation therapy have been alive for more than three years so far. The other two patients who did not receive adjuvant therapy died within one year. One patient with stage IVA died ten years after primary debulking surgery and adjuvant chemotherapy.

The clinical characteristics and surgical outcomes were compared between the young-old and old-old groups (Table 3). The old-old group had significantly more patients with ASA class 3 than the young-old group (*p* = 0.016). Although the preoperative hemoglobin level was significantly lower in the old-old group than in the young-old group (*p* < 0.001), the postoperative hemoglobin level and transfusion rate were not significantly different between the two groups. The type or stage of gynecologic malignancy and method of surgical approach were not significantly different between the two groups. Radical hysterectomy showed no significant difference according to the age in the patients including EC and CC (*p* = 0.097). Optimal debulking surgery of OC patients was not significantly different between the two groups (*p* = 1.000). In addition, the lymphadenectomy was not significantly different between the two groups (*p* = 0.172). The time to recovery from surgery was not significantly different between the two groups. The mean durations from surgery to liquid diet were 3.2 days and 3.7 days in the young-old and old-old groups, respectively (*p* = 0.292). The durations of hospital stay were 10.0 days and 11.2 days in the young-old and old-old groups, respectively (*p* = 0.254). Postoperative complications occurred in 19 (14.5%) patients, including 13 in the young-old group and 6 in the old-old group. There was no significant difference between the two groups (*p* = 0.202). The most common complication was ileus, which was resolved with conservative management. Interventions for complications were required in 8 patients. There were 4 wound repairs, 1 double J-stent placement, 1 L-tube placement, 1 colostomy, and 1 percutaneous catheter drainage insertion.

The mean follow-up periods were 38.1, 35.5, and 60.2 months for the OC, EC, and CC groups, respectively. The numbers of patients who died were 14 in the OC group and 5 in the EC group, and there were no deaths in the CC group. The overall survival rates in the OC, EC, and CC groups were 68%, 86%, and 100%, respectively. The survival rate was not significantly different between the young-old and old-old groups (*p* = 0.206). According to the cancer type, the survival rates were not significantly different between the age groups (OC: *p* = 0.630, EC: *p* = 0.279).

Of the 131 older patients, 4 died within 6 months after surgery (Table 4). Patient 1 was a 67-year-old patient with stage IIIC OC and clear-cell carcinoma. At the time of OC diagnosis, invasive ductal carcinoma of the breast was diagnosed concurrently. During surgery, severe abdominal adhesions were found, and only bilateral salpingo-oophorectomy and multiple biopsies were performed. Adjuvant treatment was discontinued after 2 cycles of chemotherapy because of the patient’s deteriorated general condition. She died just 14 days after the second round of chemotherapy. Patient 2 was 71 years old with stage IIIB EC and clear-cell carcinoma who underwent adjuvant radiotherapy after surgery. Although a partial response was observed, she was diagnosed with lung metastasis, pneumonia, and bacterial peritonitis, and died 141 days after surgery. Patient 3 was a 74-year-old woman with stage IVB EC and a malignant mixed Müllerian tumor. She underwent total hysterectomy and bilateral salpingo-oophorectomy. She refused further treatment after 1 cycle of adjuvant chemotherapy because of the side effects, including nausea and epigastric pain. The patient died 130 days after surgery because of disease progression. Patient 4 was 83 years old with stage IIIC OC and high-grade serous carcinoma. She underwent debulking surgery but died on postoperative day 3 because of asphyxia caused by bile vomiting.

## 4. Discussion

### 4.1. Main Findings

The aging population is progressing worldwide, and the age of patients with gynecologic cancer is expected to increase. In the current study, we analyzed the surgical outcomes and overall survival of patients with gynecologic cancer, aged ≥65 years. Although the old-old group had lower hemoglobin levels and worse ASA scores before surgery than the young-old group, there were no differences in the rate of blood transfusion, recovery time to diet, and hospital stay after surgery between the groups. The types of surgery including radical hysterectomy, optimal debulking surgery, and lymphadenectomy did not show significant differences between the groups. Most surgical complications were tolerable in the older patients and did not differ between the young-old and old-old groups. In addition, the survival rate did not show a significant decrease in either of the age groups. Most previous studies have reported that gynecologic cancer in older women is well tolerated; however, it is associated with less radical surgery and increased complication rates.

### 4.2. Ovarian Cancer in Older Patients

Similar to our results, Trillsch et al. reported a prospective study of 275 patients with OC undergoing cytoreductive surgery [15]. The rate of intraoperative complications, including bladder, liver, or spleen lesions, cardiac ischemia, large vessel laceration, and mass transfusions, was comparable in patients with OC aged <70 years and ≥70 years (*p* = 0.532). Postoperative complications were also not significantly different between age groups (36% and 27.7%, respectively; *p* = 0.495). Although older patients aged ≥70 years often received less radical treatment and had a higher rate of suboptimal surgical outcomes than younger patients, age itself did not show a consistent prognostic effect. Van Walree et al. reported similar complication rates according to age (<75 years: 43%, ≥75 years: 44%, *p* = 0.93) in patients with OC who underwent cytoreductive surgery [11]. In contrast, previous studies have reported different complication rates according to age. Aletti et al. compared patients aged <75 years and ≥75 years with stages IIIC–IV OC [16]. Age and performance status were the major predictors of morbidity, such as readmission, reoperation, thromboembolism, major cardiac events, pneumonia, and sepsis, in the first 30 days after surgery. Multivariate analysis revealed that age and residual disease were significantly associated with overall survival. Because of the survival benefit from optimal debulking in OC, less aggressive surgical treatment results in worse overall survival. A study comparing patients aged ≥70 and <70 years with stages I–IV OC found that residual disease and performance status affected survival, but age was not a significant factor [17]. The optimal debulking rate was 60.2%, and the 5-year survival rates were 57.8% and 56.2% in the younger and older groups, respectively. Although the mean age of patients with OC in the current study was 71 years, the optimal debulking was achieved in 67.9% of the patients, similar to findings of previous reports that were performed in the general age group [18,19]. Additionally, lymphadenectomy was commonly performed without increasing related complications in older patients with gynecologic cancer (62.3% in OC, 75.0% in EC, and 66.7% in CC). In a previous study comparing surgical outcomes with and without lymphadenectomy in patients with OC and EC aged >70 years, there were no significant differences in transfusion, postoperative complications, and hospital stay between the groups, except for the long operative time in the lymphadenectomy group [20].

### 4.3. Endometrial Cancer in Older Patients

A study of 25,698 women aged ≥65 years with EC who underwent hysterectomy showed no differences in the rates of intraoperative complications based on age [21]. However, the rate of lymphadenectomy decreased with age from 56.3% in women aged 66–69 years to 42.9% in women aged ≥85 years (*p* < 0.0001). The perioperative mortality rates were 0.4% in women who were aged 65–69 years and 1.6% in those aged ≥85 years. Although the perioperative mortality rate was 4-fold higher in the oldest women, the median hospital stay was 2 days longer in women aged ≥85 years than in those aged 65–69 years.

In another study of 124 patients with EC aged ≥65 years, there was no difference in disease-specific survival after surgery between those aged ≥75 years and those aged <75 years (78% and 82%, respectively; *p* = not significant) [22]. None of the patients died during the perioperative period. Although the rate of perioperative complications was 13.1% and the complication rate was significantly increased in patients aged ≥75 years (<75 years: 9.3%, ≥75 years: 23.1%; *p* = 0.032), there was no significant difference in the duration of hospital stay between the two groups. In the logistic regression model, age ≥75 years (*p* = 0.016, odds ratio [OR] = 4.88), chronic lung disease (*p* = 0.043, OR = 5.88), and lymphadenectomy (*p* = 0.034, OR = 4.87) were associated with high perioperative morbidities.

Fleming et al. compared patients aged 50–69 years and those aged 70–92 years with stages IA–IIB endometrioid endometrial adenocarcinoma and reported that age was not an independent poor prognostic factor for survival in multivariable analysis [23]. There was no difference between the two groups in the rates of lymphadenectomy and adjuvant therapy among all stages. The study showed that advanced age of ≥70 years was not a poor prognostic factor for overall survival in early-stage EC with the endometrioid histologic subtype. Non-endometrioid histology in EC has aggressive characteristics, with an incidence of approximately 10% in older patients [24,25]. Older patients are more likely to have non-endometrioid and poorly differentiated histology [12,13]. In the present study, the incidence of non-endometrioid histology was 38.6%. Two patients with EC died within 6 months after surgery. All the patients had non-endometrioid types, including a clear-cell and malignant mixed Müllerian tumor, and died because of disease progression.

### 4.4. Cervical Cancer in Older Patients

Radical hysterectomy, a standard treatment for early CC, is usually associated with a high rate of complications [26]. George et al. compared the postoperative morbidity and morbidity after radical hysterectomy in 8199 patients with CC according to age group [27]. Compared to young women (<50 years), older women (>70 years) were more likely to have any complications, including intraoperative, surgical site, and medical complications (22.1% versus [vs.] 34.9%). However, similar complication rates were observed in older patients aged 60–69 years (31.4%) and those older than 70 years of age (34.9%). In a retrospective study by Fuchtner et al., radical surgery-related complications were not significantly different between patients aged ≤65 and >65 years (9.9% and 11.1%, respectively; *p* = 0.825) [28]. The results showed that age alone is not a contraindication for radical hysterectomy in older patients. However, according to a report by Eggemann et al., the proportion of indicated but not performed treatment proportionally increased with the age of the patients, and older women with CC were more likely undertreated than their younger counterparts [29].

Primary radiation therapy is an alternative non-surgical treatment strategy for patients with CC. In a study of older patients with stages Ib–IV CC, similar rates of adverse effects were caused by surgery or radiotherapy according to age groups (24.3% in <65 years, 25.0% in ≥65 years; *p* = 1.000) [30]. Older patients tended to receive less standard treatment than younger patients at any disease stage (*p* < 0.001). Although the 5-year overall survival of all clinical stages was shorter in older patients than in younger patients (74.7% vs. 57.1%, *p* < 0.001), age was not an independent prognostic factor. In the multivariate analyses, the clinical stage, histology, primary surgery, and treatment intensity were independent prognostic factors.

Herein, surgical outcomes and overall survival in gynecologic cancer patients aged ≥65 years were found to be excellent in both young-old and old-old patients. The result of this study showed that overall survival rates in the OC, EC, and CC groups were 68%, 86%, and 100%, respectively. The survival rate was not significantly different between the young-old and old-old groups (*p* = 0.206). The factors suspected to affect postoperative outcomes are not only age, but also advanced stage and worse comorbidity score [31,32]. In a study comparing ASA classes 1–2 and 3–4 with gynecologic oncologic patients aged ≥70 years, the postoperative complications, including infectious morbidity and surgical wound problems, were not significantly different between ASA classes 1–2 and 3–4 (40% and 39% and 14% and 15%, respectively) [33]. Severe cardiovascular and pulmonary morbidities were significantly increased in ASA classes 3–4 (16% and 2%, respectively). In the present study, there were no patients with ASA class 4, and it seems that patients with more favorable comorbidities were included in our study. The complications commonly found in this study were wound and urinary complications; severe cardiovascular and pulmonary complications were not observed. Therefore, it is necessary to establish an optimal treatment strategy through comprehensive preoperative assessment, considering the biological age and functional status of patients.

Although previous studies focusing on older patients reported no additional postoperative morbidity and decreased long-term survival, surgical treatment was not adequately offered in older patients [30]. Concerns about complications associated with gynecologic cancer surgeries are substantial in older patients. Less ideal treatment in older patients is more associated with a reduced survival rate [16,30]. In addition, most clinical trials have focused on the general population, with a median age of 56–63 years, without considering additional comorbidities in older patients [34,35]. There has been an underrepresentation of older patients in clinical trials [36]. Therefore, there is a lack of information regarding proper treatment approaches in older patients, and more studies focusing on geriatric oncology are needed.

Several limitations of our study are related to the retrospective nature, including incomplete medical recording. Patients treated non-surgically were excluded, which may contribute to the selection bias. If more older patients were treated with chemotherapy or radiation rather than surgery, the selection bias might improve outcomes in the older patients. Another limitation is the single-center study with a relatively small sample size, and our incomplete understanding of other potential factors limit the interpretation of our data. Further prospective studies with long-term follow-up data are needed to establish the appropriate treatment strategies in older patients with gynecologic cancers.

## 5. Conclusions

In conclusion, older patients with gynecologic cancers showed good surgical outcomes and tolerable postoperative complications in this study. Therefore, surgical treatment can be safely performed in older patients with gynecologic cancers. Considering the increasing incidence of gynecologic cancers in older patients, further studies about geriatric oncology are needed, including randomized clinical trials that incorporate comorbidities in older patients.

## Figures and Tables

**Table 1:** Preoperative characteristics (*n* = 131).

Variables	Number of Patients (%)
Age	
65–74 years	106 (80.9)
≥75 years	25 (19.1)
BMI (kg/m^2^)	
<18.5	4 (3.1)
18.5–22.9	45 (34.4)
23–24.9	35 (26.7)
25–29.9	32 (24.4)
≥30	14 (10.7)
Past abdominal surgery	45 (34.4)
ASA class	
1	15 (11.5)
2	93 (71.0)
3	23 (17.6)
Number of major comorbidities	
0	44 (33.6)
1	51 (38.9)
2	30 (22.9)
3	5 (3.8)
4	1 (0.8)
Major comorbidities	
Hypertension	68 (51.9)
Diabetes mellitus	28 (21.4)
Previous malignancy	10 (7.6)
Cardiovascular disease	7 (5.3)
Cerebrovascular disease	5 (3.8)
Asthma	5 (3.8)
Thyroid disease	5 (3.8)
Renal disease	2 (1.5)
Type of gynecologic malignancy	
Ovarian or primary peritoneal cancer	53 (40.5)
Endometrial cancer	44 (33.6)
Cervical cancer	30 (22.9)
Leiomyosarcoma	4 (3.1)

BMI, body mass index; ASA, Anesthesiologists Physical Status.

**Table 2:** Analysis of clinical characteristics according to the gynecologic cancer type.

Variables	Ovarian Cancer (*n* = 53)	Endometrial Cancer (*n* = 44)	Cervical Cancer (*n* = 30)
Age (mean ± SD)	71.1 ± 4.9	69.9 ± 4.4	68.8 ± 3.2
65–74 years	40 (75.5)	36 (81.8)	28 (93.3)
≥75 years	13 (24.5)	8 (18.2)	2 (6.7)
FIGO stage			
I	11 (20.8)	24 (54.5)	23 (76.7)
II	7 (13.2)	6 (13.6)	4 (13.3)
III	23 (43.4)	9 (20.5)	2 (6.7)
IV	12 (22.6)	5 (11.4)	1 (3.3)
Surgical approach			
Laparoscopy	2 (3.8)	13 (29.5)	9 (30.0)
Laparotomy	51 (96.2)	25 (56.8)	19 (63.3)
Vaginal	-	6 (13.6)	2 (6.7)
Type of hysterectomy			
Simple hysterectomy	-	24 (54.5)	10 (33.3)
Radical hysterectomy	-	20 (45.5)	20 (66.7)
Lymphadenectomy	33 (62.3)	33 (75.0)	20 (66.7)
Adjuvant treatment			
None	14 (26.4)	12 (27.3)	16 (53.3)
Chemotherapy	39 (73.6)	11 (25.0)	-
Radiotherapy	-	15 (34.1)	1 (3.3)
CCRT or chemotherapy + radiotherapy	-	6 (13.6)	13 (43.3)

SD, standard deviation; FIGO, International Federation of Gynecology and Obstetrics; lymphadenectomy, pelvic and paraaortic lymphadenectomy; CCRT, concurrent chemoradiation therapy.

**Table 3:** Comparison of clinical characteristics and surgical outcomes between the young-old and old-old groups.

Variables	Young-Old (*n* = 106)	Old-Old (*n* = 25)	*p* Value
BMI (kg/m^2^)	24.8 ± 4.0	23.6 ± 3.6	0.188
ASA class			
1 or 2	92 (86.8)	16 (64.0)	0.016
3	14 (13.2)	9 (36.0)	
Number of comorbidities			
≤1	68 (64.2)	12 (48.0)	0.136
≥2	38 (35.8)	13 (52.0)	
Past abdominal surgery			
No	68 (64.2)	18 (72.0)	0.494
Yes	38 (35.8)	7 (28.0)	
Type of gynecologic malignancy			
Ovarian or primary peritoneal cancer	40 (37.7)	13 (52.0)	0.069
Endometrial cancer	36 (34.0)	8 (32.0)	
Cervical cancer	28 (26.4)	2 (8.0)	
Leiomyosarcoma	2 (1.9)	2 (8.0)	
FIGO stage			
I	48 (45.3)	11 (44.0)	0.962
II	14 (13.2)	3 (12.0)	
III	28 (26.4)	8 (32.0)	
IV	16 (15.1)	3 (12.0)	
Surgical approach			
Laparoscopy	21 (19.8)	3 (12.0)	0.237
Laparotomy	77 (72.6)	22 (88.0)	
Vaginal	8 (7.5)	0	
Type of hysterectomy ^†^			
Simple hysterectomy	32/64 (50.0)	2/10 (20.0)	0.097
Radical hysterectomy	32/64 (50.0)	8/10 (80.0)	
Optimal debulking ^‡^			
No	13/40 (32.5)	4/13 (30.8)	1.000
Yes	27/40 (67.5)	9/13 (69.2)	
Lymphadenectomy			
No	35 (33.0)	12 (48.0)	0.172
Yes	71 (67.0)	13 (52.0)	
Operation time (min)	239.1 ± 131.9	239.7 ± 105.3	0.981
EBL (mL)	806.6 ± 748.3	900.0 ± 1043.6	0.620
Preoperative hemoglobin (g/dL)	12.4 ± 1.3	11.2 ± 1.6	<0.001
Postoperative hemoglobin (g/dL)	10.6 ± 1.4	10.7 ± 1.8	0.735
Intra- or postoperative blood transfusion			
No	55 (51.9)	9 (36.0)	0.153
Yes	51 (48.1)	16 (64.0)	
Duration from surgery to liquid diet (day)	3.2 ± 1.8	3.7 ± 1.9	0.292
Hospital stay (days)	10.0 ± 5.2	11.2 ± 4.3	0.254
Postoperative complications			
None	93 (87.7)	19 (76.0)	0.202
Ye	13 (12.3)	6 (24.0)	
Ileus	7	2	
Sepsis	1	0	
Wound complication	4	1	
Urinary retention	0	1	
Hydronephrosis	2	0	
Enterovaginal fistula	1	0	
Lymphocysts	1	0	
Herpes zoster	0	1	
Interventions to manage complications			
None	6 (46.2)	5 (83.3)	0.177
Yes	7 (53.8)	1 (16.7)	
Wound repair	3	1	
Double J-stent placement	1	0	
L-tube placement	1	0	
Colostomy	1	0	
PCD insertion	1	0	

^†^ Type of hysterectomy included patients with endometrial and cervical cancer. ^‡^ Optimal debulking included indicated for patients with ovarian cancer. BMI, body mass index; ASA, Anesthesiologists Physical Status; EBL, estimated blood loss; PCD; percutaneous catheter drainage.

**Table 4:** Analysis of patients who died within 6 months of surgery.

	Patient 1	Patient 2	Patient 3	Patient 4
Age	67	71	74	83
BMI (kg/m^2^)	17.1	17.6	22.2	21.5
ASA class	2	3	2	2
Comorbidity	Breast cancer	Tuberculous spondylitis	HTN	HTN
Cancer origin	Ovary	Endometrium	Endometrium	Ovary
Histologic type	Clear cell	Clear cell	MMMT	High-grade serous
Stage	IIIC	IIIB	IVB	IIIC
Operation	BSO, pelvic peritoneum biopsy, mastectomy	TAH, BSO, LND, omentectomy	TAH, BSO	TAH, BSO, LND, omentectomy, appendectomy, diaphragm ablation, pelvic peritonectomy
Packed RBC transfusion (unit)	2	2	0	5
Postoperative complication	Cellulitis	None	None	None
Adjuvant treatment	Chemotherapy 2 cycle	Radiotherapy 5040cGy	Chemotherapy 1 cycle	None
Duration from surgery to death (day)	54	141	130	3
Cause of death	Disease progression	Disease progression	Disease progression	Asphyxia

BMI, body mass index; ASA, Anesthesiologists Physical Status; HTN, hypertension; MMMT, malignant mixed Mullerian tumor; BSO, bilateral salpingo-oophorectomy; TAH, total abdominal hysterectomy; LND, lymph node dissection.

## Data Availability

All the studies used in this study are published in the literature.
